# Epidemiology of HCV and HBV in a High Endemic Area of Southern Italy: Opportunities from the COVID-19 Pandemic—Standardized National Screening or One Tailored to Local Epidemiology?

**DOI:** 10.3390/biology11040609

**Published:** 2022-04-16

**Authors:** Riccardo Nevola, Vincenzo Messina, Aldo Marrone, Nicola Coppola, Carolina Rescigno, Vincenzo Esposito, Vincenzo Sangiovanni, Ernesto Claar, Mariantonietta Pisaturo, Francesco Maria Fusco, Pietro Rosario, Antonio Izzi, Raffaella Pisapia, Valerio Rosato, Paolo Maggi, Luigi Elio Adinolfi

**Affiliations:** 1 Internal Medicine Unit, Department of Advanced Medical and Surgical Sciences, University of Campania “Luigi Vanvitelli”, 80138 Naples, Italy; aldo.marrone@unicampania.it (A.M.); luigielio.adinolfi@unicampania.it (L.E.A.); 2 Hepatology Unit, Ospedale Evangelico Betania, 80147 Naples, Italy; ernestoclaar@gmail.com (E.C.); valeriorosato@gmail.com (V.R.); 3 Infectious Diseases Unit, AORN Sant’Anna e San Sebastiano, 81100 Caserta, Italy; vincenzomessina.ce@libero.it (V.M.); paolo.maggi@unicampania.it (P.M.); 4 Infectious Diseases Unit, Department of Mental Health and Public Medicine, University of Campania “Luigi Vanvitelli”, 80138 Naples, Italy; nicola.coppola@unicampania.it (N.C.); mariantonietta.pisaturo@unicampania.it (M.P.); 5 Infectious Diseases and Neurology Unit, Cotugno Hospital, 80131 Naples, Italy; carolinarescigno@libero.it (C.R.); izziantonio@yahoo.it (A.I.); raffaellapisapia@gmail.com (R.P.); 6 IVth Division of Immunodeficiency and Gender Infectious Diseases, Cotugno Hospital, 80131 Naples, Italy; vincenzoesposito@ospedalideicolli.it (V.E.); pietro.rosario@ospedalideicolli.it (P.R.); 7 IIIrd Infectious Diseases Unit, Cotugno Hospital, 80131 Naples, Italy; vincenzo.sangiovanni@ospedalideicolli.it (V.S.); francescomaria.fusco@ospedalideicolli.it (F.M.F.)

**Keywords:** HCV, HBV, screening program, epidemiology, COVID-19

## Abstract

**Simple Summary:**

Epidemiological data on viral hepatitis are essential to optimize screening programs. For HCV, the Italian Health Ministry planned a cohort screening for those born in 1969–1989. In order to update the epidemiological data of viral hepatitis in a highly endemic area of Southern Italy and assess whether the screening programs currently planned by the Italian government for those born between 1969–1989 will be effective, a retrospective multicenter study was carried out enrolling all COVID-19 hospitalized patients screened for markers of HCV and HBV infection. Indeed, the COVID-19 pandemic has resulted in access to the national health system of an unselected population similar to the general one. Among the 2126 patients evaluated, HBsAg and HCV-Ab prevalence was 1.6% and 5.1%, respectively. For HCV infection, a bimodal distribution was observed, with peaks in the birth cohorts 1930–1939 and 1960–1969 (11.6% and 5.6%, respectively). An analysis of the screening period imposed (born: 1969–1989) demonstrates that only 17% of HCV infection could be captured. Thus, an alignment of the screening period (i.e., birth cohort 1960–1984) would capture 40% of cases. Data show a high endemicity of hepatitis virus in our geographic area and the need for a tailored regional screening program.

**Abstract:**

The COVID-19 pandemic led to the hospitalization of an unselected population with the possibility to evaluate the epidemiology of viral hepatitis. Thus, a retrospective multicenter study was conducted in an area of Southern Italy with the aim of assessing the prevalence of HCV and HBV markers and the ability of current screening program to capture cases. We evaluated 2126 hospitalized patients in seven COVID Centers of Naples and Caserta area in which 70% of the Campania population lives. HBsAg and HCV-Ab prevalence was 1.6% and 5.1%, respectively, with no differences between gender. Decade distribution for birth year shows a bimodal trend of HCV prevalence, with a peak (11.6%) in the decade 1930–1939 and a second peak (5.6%) for those born in 1960–1969. An analysis of the screening period imposed by the Italian government for those born between 1969 and 1989 shows that only 17% of cases of HCV infection could be captured. A small alignment of the screening period, i.e., those born from 1960 to 1984, would capture 40% of cases. The data confirm the high endemicity of our geographical area for hepatitis virus infections and underline the need for a tailored screening program according to the regional epidemiology.

## 1. Introduction

Viral hepatitis is still a major global public health issue to date. Although the incidence of non-alcoholic fatty liver disease (NAFLD) is constantly increasing, major hepatotropic viruses still represent the main cause of liver cirrhosis and hepatocellular carcinoma (HCC) [1]. Vaccination against hepatitis B virus (HBV), the availability of effective antiviral therapy for the suppression of HBV viremia, or clearance of hepatitis C virus (HCV) infection by direct antiviral agents (DAA), significantly reduced the spread of viral hepatitis and the rates of chronic hepatitis and liver cirrhosis, as well as the development of hepatic and extrahepatic complications [2,3,4].

At present, the real prevalence and distribution of these viral infections in Italy is not yet known and this condition represents a limit for programs aimed at their elimination. For chronic HCV hepatitis, the goal set for its elimination by the World Health Organization (WHO) is by 2030, but it seems far from being achieved to date [5]. Italy appeared to be on track to meet its HCV elimination targets in 2018, but there was a significant decline in anti-HCV antiviral treatments in 2019, due to the exhaustion of known or national health related cases [6,7]. In subsequent years, the COVID-19 pandemic further reduced anti-HCV treatments and slowing WHO program [5].

It is estimated that the decline in antiviral treatments in Italy may lead to a delay of about seven years in achieving the aim of the WHO [7]. It has been estimated that one year of delay in the virus elimination agenda could result in an overall reduction of more than 750,000 treatments for HCV with a consequence of an excess of 44,800 cases of HCC and 72,300 deaths for liver disease at the end of the year 2030 [8]. It is, therefore, necessary to resume the march towards the elimination of hepatitis viruses indicated by the WHO as soon as possible.

At present, the challenge for eliminating viral hepatitis appears to be identifying patients to treat. Therefore, together with awareness programs, screening projects for populations at risk appear to be crucial. However, in European scenarios, where prevalence rates of hepatitis virus appear low (<2%) [9], mass screening is not considered convenient, and therefore not recommended [10]. To optimize screening, it is necessary to identify the population groups at greatest risk of infection. In this context, in addition to the population at already known risk, such as people who use drugs (PWUD) and prisoners, the screening must include specific age groups at greatest risk of HBV and/or HCV infection considering the different ways of virus spreading over the decades. In this regard, it is known that the prevalence of viral hepatitis in Italy is characterized by a cohort effect, with a decreasing risk with more recent generations [11,12].

In Italy, the Ministry of Health for the two-year period 2020–2021 has provided for a cohort screening for subjects born between 1969 and 1989, as well as for prisoners and those enrolled in the public services for addiction (SerD) [13]. However, the COVID-19 pandemic has delayed the start of this screening program and there is a need to resume it. However, it should be emphasized that most of the epidemiological studies available on the prevalence of viral hepatitis in Italy were conducted over 20 years ago and therefore may not necessarily reflect the current situation [14,15]. An update on the prevalence of HCV and HBV infections in the general population is therefore needed.

The SARS-CoV-2 pandemic has resulted in the access to hospital facilities of an unselected adult population cohort mostly over 30 years old. Thus, the prevalence of HCV and HBV infections in hospitalized COVID-19 patients could represent an estimate of the distribution of these viral infections in the general population considering their diffusion in Italy [11,12].

The aim of the study was to evaluate the current prevalence and distribution of HCV and/or HBV infection in a metropolitan area of Southern Italy in patients hospitalized for SARS-CoV-2 infection and to define the ability of the current Italian screening program to capture positive cases in the context of a highly endemic region for these hepatitis viruses.

## 2. Materials and Methods

### 2.1. Study Design

A multi-center retrospective study was planned, enrolling all COVID-19 patients admitted to COVID Centers between February 2020 and May 2021. Seven Centers in the metropolitan area of Naples (3 at the Cotugno Hospital for infectious diseases; 2 at the Vanvitelli University Hospital; the Evangelico Betania Hospital) and a center in the Caserta area (Sant’Anna and San Sebastiano Hospital) participated in the study.

### 2.2. Subjects

All COVID-19 patients that were screened for serological markers for HBV and HCV-Ab were included in the study. Demographic data, such as age and gender and characteristics of screened patients, were collected.

### 2.3. Regional Context of the Study

Considering the regional distribution of beds reserved for COVID-19 patients and the catchment area of the hospitals involved in the study, our population is representative of approximately 70% of the resident population of the Campania Region (inhabitants of the province of Naples: 2.9 million, inhabitants of the province of Caserta: 0.9 million), which is 5.6 million inhabitants.

### 2.4. Laboratory Evaluation

Serum evaluation of HCV-Ab and HBsAg was performed by commercial ELISA assays.

### 2.5. Data Analysis

Age is represented as the median and interquartile range. The distribution of the prevalence of seropositivity was grouped by decade. The significance of differences in positivity proportions between groups was assessed by chi-squared test with Yates’s correction. A *p*-value < 0.05 was considered statistically significant.

## 3. Results

### 3.1. General Characteristics of the Population

During the study period, 2361 patients with COVID-19 were admitted to the COVID centers involved in the study. Of these hospitalized patients, 2126 (90%) were screened for HBV and HCV-Ab.

Table 1 shows the demographic characteristics of the screened population for each individual center. The mean age of the screened population was 62 years (IQR 50–73) and 61.8% were male. In the population studied, the number of active PWUDs was low (0.75%) and there were no prisoners. More than 99% of them were of Caucasian origin.

### 3.2. HCV-Ab Screening

**Figure 1:**
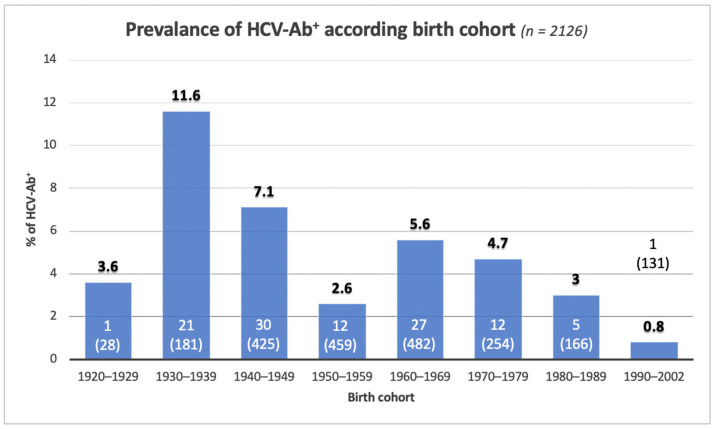
HCV-Ab seroprevalence, according to the birth cohort. In columns: numbers in parenthesis indicate the total number of screened subjects; number not in parenthesis indicate the number of positive cases.

Of 2126 subjects screened for HCV-Ab, 109 were positive, therefore the prevalence rate of HCV infection in the population studied was 5.1%. Figure 1 shows the distribution of HCV-Ab prevalence in the birth cohorts of subjects divided over decades. The prevalence rates of HCV-Ab show an increasing trend in relation to age. The highest rates are observed among patients born between 1930 and 1939 (11.6%) followed by those born between 1940 and 1949 (7.1%). A significant reduction in prevalence rates is observed in those born between 1950 and 1959 (2.6%; *p* < 0.001), while in those born between 1960 and 1969 a significant new increase in prevalence rates was observed (5.6%; *p* < 0.021). In those born from 1970 to 2002, a steady progressive reduction in HCV infection was observed, reaching a prevalence of 0.76% in the 1990–2002 cohort.

**Figure 2:**
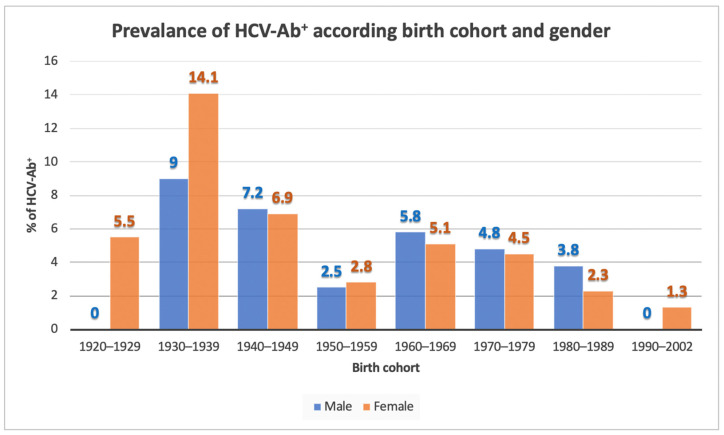
HCV-Ab seroprevalence, according to the birth cohort and gender.

Figure 2 reports the prevalence rates of HCV Ab positivity in relation to gender and birth cohort. The data show that there is no significant difference in gender-related prevalence rates (5.4% in females, 4.9% in males-*p* = 0.713).

### 3.3. HBV Screening

**Figure 3:**
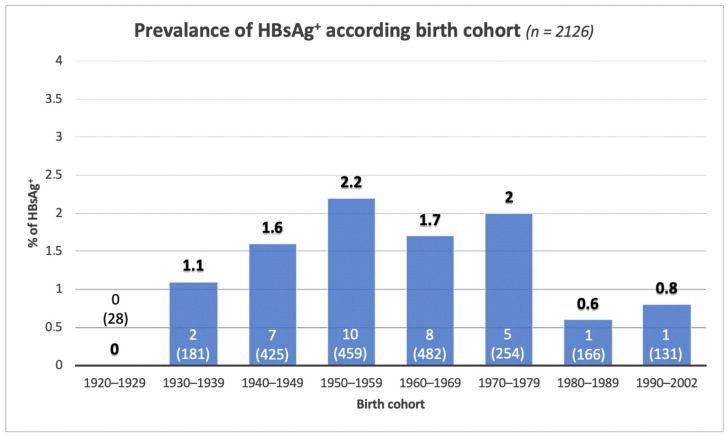
HBsAg seroprevalence, according to the birth cohort. In columns: numbers in parenthesis indicate the total number of screened subjects; number not in parenthesis indicate the number of positive cases.

Of the 2126 subjects tested, 34 were positive for HBsAg for an overall prevalence of 1.6%. Figure 3 shows the prevalence of HBsAg by birth cohort. Prevalence rates range from 0.6%, in those born between 1980 and 1989, to 2.2% in those born between 1950 and 1959. A significant reduction in the incidence of HBV infection has been observed since 1980. No cases have been identified in those born before 1930 or after 1995, although the number of patients screened was low.

**Figure 4:**
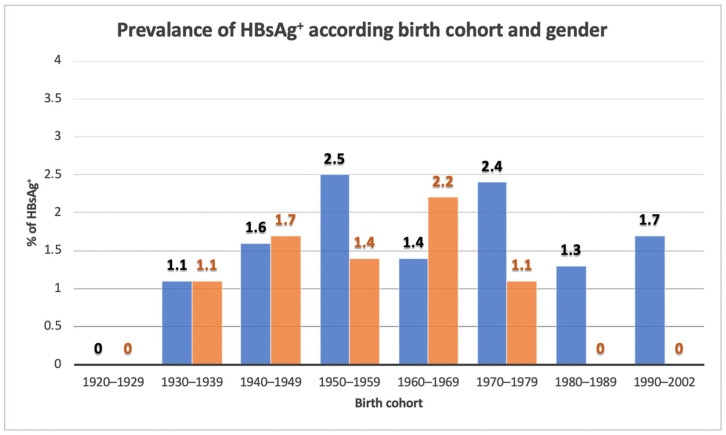
HBsAg seroprevalence, according to the birth cohort and gender.

Figure 4 reports the prevalence rates of HBsAg positivity expressed by gender and birth cohort. No significant differences between males (1.8%) and females (1.2%; *p* = 0.373) were observed.

### 3.4. Co-Infection HBV/HCV

Four cases of HBV/HCV coinfection (0.2%) were detected in screened patients. Of these, 3 patients were male.

### 3.5. Distribution of HCV-Ab Accordingly to Current Italian Health Ministry Screening Program

The program of the Italian Health Ministry provides for a screening for HCV-Ab of subjects born between 1969 and 1989 [13]. In our study, in this birth cohort, 467 subjects were evaluated for HCV-Ab, of which 19 (4.1%) were found to be serum positive. Thus, considering that the number of HCV-Ab positive in the whole evaluated cohort was 109 subjects, the ability to capture HCV subjects in the screening cohort 1969–1989 was of 17.4%. In particular, on 77 subjects evaluated, who were born in the five-year period 1985–1989, no one was found to be positive for HCV-Ab, thus showing a prevalence similar to the subjects born in the following decade in which a significant fall in the prevalence of HCV infection was observed (Figure 1).

**Figure 5:**
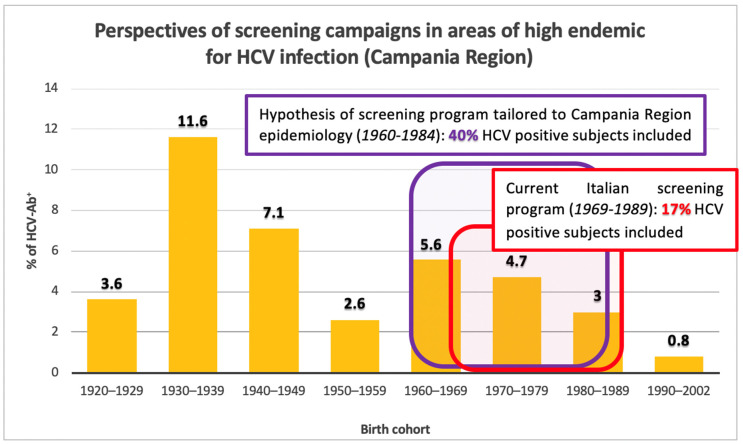
Perspectives of screening campaigns in areas of high endemic for HCV infection.

As shown in Figure 1, in the Campania Region, an epidemiological peak began with those born in 1960 with a drastic fall starting from 1985. The subjects born between 1960 and 1984 included in our study were 825 of which 44 (5.3%) were found to be HCV-Ab positive. On this basis, the ability to capture HCV-Ab positive subjects in the 1960–1984 born cohort is 40%. Therefore, a serological assessment for HCV in the 1960–1984 cohort versus the 1969–1989 cohort would significantly increase screening efficiency by detecting 40% of positive subjects versus 17% (*p* < 0.003) (Figure 5).

Birth cohort screening programs should be understood as part of a larger screening program that includes other categories at high risk (e.g., PWUD, prisoners, hospitalized patients).

## 4. Discussion

Large studies indicate that the prevalence rates of HCV seropositivity in Italy is 1.1% and for HBV it is less than 1% [9,16]. However, other studies estimate higher rates [17,18]. Mancusi et al. [17] in a meta-analysis hypothesize national prevalence rates of HCV infection of 2.6%. Similarly, Andriulli et al. [18] has reported a prevalence of HCV-Ab of 2.3%. Many studies have shown an increasing North–South gradient [17,19,20].

Our study, conducted in Southern Italy, in an area considered to be of high prevalence for hepatitis viruses, shows an overall rate of seropositivity for HCV-Ab of 5.1%. The data are in line with other reports of the metropolitan area of Naples, the Campania region and other areas of southern Italy [11,21]. Fusco et al. [22] showed a prevalence of HCV positive subjects of 7.5% in the Neapolitan population with an age-related gradient. More recently, Morisco et al. [23] estimated a 3% prevalence of HCV-Ab positive in the same metropolitan area of our study, with a peak of 8.2% in those born between 1945 and 1955. In the latter birth cohort, our data confirm a high prevalence rate (5.5%), even if the peak we highlighted emerges in particular in those born between 1930 and 1939 (11.6%). In another area of Southern Italy (Calabria Region), Guadagnino et al. had already estimated a prevalence rate of HCV-Ab positivity of 5.7%, which appeared to be in sharp decline when compared to the data available in the same area 14 years earlier (12.6%) [11]. In our cohort, we showed a prevalence (5.1%) higher than that reported in studies from the North (1%) [17,24] and in Central Italy (1.9–4.1%) [17,25,26], confirming the already known North–South increasing gradient [17,19,20].

Overall, our study shows that the prevalence of HCV-Ab positive subjects increases with age, similar to what emerged from other studies performed on the general population [11,12,15,18,20,22,23,27,28,29]. In particular, in our study, the prevalence curve shows a bimodal distribution, with a first peak in subjects born between 1930 and 1939 and the second one in those born between 1960 and 1969 (Figure 1). Previously, Polilli et al. [26] identified on a large population of the Abruzzo Region (Central Italy) two prevalence peaks similar to those observed in our population. A similar distribution of HCV-Ab emerged also in Northern Italy [30], underlining a national homogeneity in the history of the virus spread.

The first peak of HCV-Ab prevalence observed in this study probably reflect the iatrogenic exposure secondary to the use of reusable glass syringes (typically used in the 1950s and 1960s for the polio vaccine and for other intramuscular injections) and, subsequently, to the spread of intra-family infection [12,21,23,28]. This method of diffusion finds analogies with what occurred in Egypt for parenteral therapy against schistosomiasis [31]. Due to the characteristics of the population (advanced age, higher mortality, and morbidity rates for patients with HCV infection compared to HCV-negative patients), this endemic wave has the tendency to extinguish [11], although it still represents a significant reservoir for HCV and a reason for possible virus spreading.

It is also interesting to note that, among subjects born before 1930, the prevalence curve drops sharply, probably as a consequence of the survival effect, with a probable higher mortality of patients with chronic HCV infection compared to seronegative patients, as already showed by previous studies [23,32]. Comparison with the data of Guadagnino et al. [15] relating to the late 1990s confirms this hypothesis. In the population they examined, the prevalence of HCV-Ab in the over-60s (born approximately before 1935) was 33%, significantly higher than what we found for similar birth cohorts (11%), underlining the likely greater mortality of the HCV-positive cohort. Therefore, the first and most consistent wave of HCV spread in Italy (1950s–1960s) due to iatrogenic diffusion is naturally fading away [32,33].

Our study shows that the second HCV-Ab prevalence peak is evident in subjects born between 1960 and 1969 (5.6%, Figure 1), with a progressive decrease towards more recent birth cohorts. This endemic wave is probably due to the boom in the use of intravenous drugs that occurred in the 1980s [7,21].

As regards the distribution between genders, the prevalence of HCV-Ab appears not significantly different between women and men (5.4% vs. 4.9%, respectively–*p* = 0.713). Data available on the gender distribution of HCV infection appear to be conflicting, overall, without a clear predominance of one gender over the other [18,22,25,29,34].

In analogy with what was observed for HCV infection, in our study also the prevalence of HBV infection was found to be higher in our geographical area (1.6%) than the national average. Our data are in line with those previously shown by Fusco et al. (2.2%) on a population belonging to the same metropolitan area [22]. The reported prevalence of HBV in northern Italy is around 1% [30] and the estimated national prevalence is also less than 1% [16]. Our data and those of Fusco [22] indicate that the prevalence rate of HBV infection in the metropolitan area of Naples is also higher than that reported in other regions of southern Italy, such as Sicily, where HBsAg prevalence was of 0.7% [28].

Differently from HCV infection, the HBsAg positivity rate does not show a clear increasing trend with age. However, similarly to what was estimated by previous studies [22], the peak prevalence is reached in those born between 1950 and 1959 (2.2%), and then progressively declines among those born before 1950. It is not known whether this demographic trend is due to a survival effect (higher mortality among HBV positive elderly patients) or to a subsequent viraemic wave.

Our data show a similar prevalence of HBV infection in males (1.8%) and women (1.2% in women, *p* = 0.373). Unlike our data, Fusco et al. [22] showed in the same geographical area (Naples and Campania Region) fewer women than men being HBsAg-positive (OR = 0.6).

Overall, our data collected on a representative population of a large and populous area of Southern Italy show prevalence rates of viral hepatitis significantly higher than the national average. In this context, the SARS-CoV-2 pandemic must no longer be considered as an obstacle to the global elimination of viral hepatitis, but as an opportunity. As already advocated by other authors [27,35], the access to health systems (hospital admissions, vaccinations, mass tests for SARS-CoV-2 research) of large population groups imposed by the COVID-19 pandemic must be seen as an opportunity for screening and linkage to care for the diagnosis and treatment of viral hepatitis and to reverse the tendency of decreasing antiviral treatments prior to the pandemic. Giacomelli et al. [27] proved that this is a winning model. The combination of a free serological screening for SARS-CoV-2 and HCV in an area of Northern Italy made it possible to identify the seropositivity for HCV and the subsequent linkage to care of 72 patients (out of 2505 tested, 2.9%), of whom less than half were previously aware of their status. Therefore, according to Crespo et al. [35], it would be desirable to perform the antibody screening test for viral hepatitis (HBV and HCV) in all patients who undergo testing for SARS-CoV-2.

The HCV infection screening imposed by the Italian government at present seems to be an important tool, but potentially insufficient in order to eradicate the virus. Regardless of the modalities in which it will be carried out and the population adherence, this screening program (free first-level tests for the entire population born between 1969 and 1989, for prisoners and members of the SerD [13]) aims to be cost-effective, focusing resources on diagnosis (and subsequent treatment) in a young population at high risk of HCV infection and with a likely high rate of previously undiagnosed cases [7]. However, this national standardized screening program does not consider the significant differences in local epidemiology and does not include older population groups (e.g., born before 1950) who show higher prevalence rates than the currently target birth cohorts (up to 11.6% vs. 4.1% of those born between 1969 and 1989) and who are at greater risk of virus spread and circulation, in addition to being more vulnerable due to age and comorbidities. Moreover, in the advanced stages of the disease (prevalent prerogative of older age groups in relation to the longer duration of the disease), antiviral treatment with DAAs is to be considered life-saving, favorably impacting for both hepatic [36] and extrahepatic outcomes [37,38,39].

The current Italian birth cohort screening program 1969–1989 (28.7% of the Italian population, about 17 million out of 59 million people-ISTAT data [40]) would be able to include only 17% of HCV positive subjects present in a high endemic area with the same characteristics of our population (Figure 5). Moreover, the cohort of HCV positive subjects born between 1980 and 1989, which shows a lower prevalence rate compared to older age groups, appears historically linked to intravenous substance abuse and could benefit from a different screening program (e.g. SerD) than a birth cohort-based one. Previously, Kondili and the PITER (Italian Platform of the Study of Viral Hepatitis Therapies) Collaborating Group [41], on the basis of a real-life national cohort data, estimated that only a screening on more advanced age groups (1948–1978) was able to capture 75% of HCV positive patients. Furthermore, through a mathematical model, it was estimated that the extension of screening to those born between 1948 and 1967 in addition to that currently provided in Italy (1969–1989) is the most cost-effective strategy, allowing to earn approximately 144,000 quality-adjusted life years (QALYs) by 2031 and lead to a reduction of 89.3% in HCV cases [42].

In light of an updated knowledge of local epidemiology, such as that obtained from our study, in the Campania Region, few changes in the screening target population could allow a significant increase in the number of HCV positive patients identified, with a small increase in resources. Since in the Campania population the number of HCV cases appears very low among those born after 1984 (one case out of 208 in our population) and it is significantly high among those born in the 1960s, instead, (25 cases out of 435 among those born between 1960–1968, 5.7%), modifying the screening target from those born between 1969 and 1989 to the birth cohorts 1960–1984 would allow to improve the screening capture capacity from 17% up to 40% (Figure 5). In fact, if the 1969–1989 birth cohorts show a significantly higher prevalence than those born after 1984 (19 HCV-Ab positive out of 467 vs. one positive out of 208, respectively-*p* = 0.022), there is no significant difference between birth cohorts 1969–1989 and those born between 1960 and 1968 (25 HCV-Ab positive out of 435, *p* = 0.310). This advantage would be obtained at the cost of only a four-year increase in the birth cohorts involved, that in the Campania Region means an increase in the screened population of about 390,000 subjects (from 29% to 36% of the resident population) [40].

Similarly, the fitting of screening programs to local epidemiologies in all other geographical areas of Italy would probably allow to improve the screening power, maximizing the use of resources. In fact, the wide geographical heterogeneity of HCV infection rates requires a diversified screening policy, able to follow the map of the contagion.

In any case, even in our screening hypothesis, the peak prevalence of those born before 1950 would not be intercepted. However, these population groups (with greater comorbidities and then greater risk of hospitalization) could be intercepted through a systematic evaluation of infection markers at the time of access to a hospital structure. Likewise, HCV infection high risk groups (such as PWUDs and prisoners) should benefit from a dedicated screening.

Therefore, in order to achieve the objectives suggested by the WHO for 2030 [5], screening programs should be diversified on the basis of regional epidemiological data and not standardized at the national level, making homogeneous what is not. A dedicated screening should target high-risk groups instead (PWUD, prisoners, hospitalized patients).

Our study has some limitations. In particular, no data are available on either the proportion of seropositive subjects for HCV-Ab and/or HBsAg with active infection (HCV RNA and/or HBV DNA present) or co-infections with HDV and/or HIV. Furthermore, no information is available concerning the state of liver disease (severity and previously known diagnosis), about any existing or previous therapies or socio-economic conditions of patients. However, these data are beyond the scope of our study, which is meant as a first level screening aimed at quantifying the population exposed to major hepatotropic viruses and defining their distribution in relation to the birth cohort. Furthermore, although the population examined (patients with SARS-CoV-2 infection admitted to hospital facilities) is unselected and potentially comparable to the general population, some groups appear less represented (e.g., those born after 1990). Given the greater severity of SARS-CoV-2 clinical manifestations in patients with multiple morbidities, access to hospital facilities could involve a population at greater risk of viral hepatitis (e.g., patients with liver cirrhosis and/or HCC) and/or at greater risk of contagion for iatrogenic exposure (patients with multiple morbidity), possibly overestimating our data.

## 5. Conclusions

Despite the advances in recent decades, prevalence rates of viral hepatitis remain high. In the Campania Region, the current prevalence rates appear to be 1.6% for HBV infection and 5.1% for HCV-Ab; in the latter case with an overall bimodal trend increasing with age. In our highly endemic areas, HCV-Ab positive rates reach peaks up to 11.6% in individuals born before 1940. In order to maintain treatment rates able to achieve HCV elimination goals, it is necessary to improve screening programs and focus research on high prevalence groups. However, since the geographic distribution of HBV and HCV infections in Italy is not uniform, the same screening program on a national scale could appear less effective in areas of high endemicity, leading to wrongly exclude population groups characterized by high viral circulation. In order to maximize the results, it appears desirable to develop tailored screening programs according to local and regional epidemiological data.

## Figures and Tables

**Table 1:** Demographic characteristics of the screened population, overall and by single Center.

	All Patients	AOU Vanvitelli Naples		Cotugno Hospital Naples			Betania Evangelical Hospital Naples	AORN “Sant’Anna e San Sebastiano” Caserta
		Internal Medicine Unit	Infectious Diseases Unit	Infectious Diseases and Neurology Unit	IVth Division of Immunodeficiency and Gender Infectious Diseases	IIIrd Infectious Diseases Unit	Internal Medicine Unit	Infectious Diseases Unit and COVID Centers
Admitted patients, *n*	2361	208	403	207	205	219	124	995
Evaluated patients, *n* (%)	2126 (90)	191 (91.8)	390 (96.8)	201 (97.1)	179 (87.3)	138 (63.0)	109 (87.9)	918 (92.3)
Male, *n* (%)	1313 (61.8)	114 (59.7)	216 (55.4)	151 (75.1)	111 (62.0)	97 (70.3)	40 (36.7)	584 (58.7)
Age, median (IQR), y	62 (50–73)	63 (53–73)	57 (42–69)	61 (50–69)	61 (52–72)	59 (51–69)	65 (38–76)	64 (52–75)
Birth cohort								
1920–1929, *n* (%)	28 (1.3)	3 (1.6)	1 (0.3)	1 (0.5)	1 (0.9)	1 (0.7)	3 (2.7)	18 (2)
1930–1939, *n* (%)	181 (8.5)	16 (8.4)	18 (4.6)	10 (5)	13 (7.3)	7 (5.1)	11 (10.1)	106 (11.5)
1940–1949, *n* (%)	425 (20)	39 (20.4)	64 (16.4)	35 (17.4)	36 (20.1)	19 (13.8)	30 (27.5)	202 (22)
1950–1959, *n* (%)	459 (21.6)	46 (24.1)	76 (19.5)	58 (28.9)	40 (22.3)	37 (26.8)	17 (15.6)	185 (20.1)
1960–1969, *n* (%)	482 (22.7)	51 (26.7)	89 (22.8)	47 (23.4)	48 (26.8)	39 (28.3)	11 (10.1)	197 (21.5)
1970–1979, *n* (%)	254 (11.9)	22 (11.5)	51 (13.1)	30 (15.9)	23 (12.8)	25 (18.1)	7 (6.4)	96 (10.5)
1980–1989, *n* (%)	166 (7.8)	10 (5.2)	56 (14.4)	9 (4.5)	15 (8.4)	9 (6.5)	14 (12.8)	53 (5.8)
1990–2002, *n* (%)	131 (6.2)	4 (2.1)	35 (9)	11 (5.5)	3 (1.7)	1 (0.7)	16 (14.7)	61 (6.6)

## Data Availability

Data are available at request from luigielio.adinolfi@unicampania.it or riccardo.nevola@unicampania.it.
